# Cluster Analysis of Weighted Bipartite Networks: A New Copula-Based Approach

**DOI:** 10.1371/journal.pone.0109507

**Published:** 2014-10-10

**Authors:** Alessandro Chessa, Irene Crimaldi, Massimo Riccaboni, Luca Trapin

**Affiliations:** IMT Institute for Advanced Studies, Lucca, Italy; King's College London, United Kingdom

## Abstract

In this work we are interested in identifying clusters of “positional equivalent” actors, i.e. actors who play a similar role in a system. In particular, we analyze weighted bipartite networks that describes the relationships between actors on one side and features or traits on the other, together with the intensity level to which actors show their features. We develop a methodological approach that takes into account the underlying multivariate dependence among groups of actors. The idea is that positions in a network could be defined on the basis of the similar intensity levels that the actors exhibit in expressing some features, instead of just considering relationships that actors hold with each others. Moreover, we propose a new clustering procedure that exploits the potentiality of copula functions, a mathematical instrument for the modelization of the stochastic dependence structure. Our clustering algorithm can be applied both to binary and real-valued matrices. We validate it with simulations and applications to real-world data.

## Introduction

In the last few years, network theory has attracted the interest of a widespread audience as a powerful tool to model and analyse complex relationship structures. In particular, the identification of network communities, known as cluster analysis, plays a central role and it represents an active field of research (e.g. [1], [2], [3] and [4]). *Community detection* allows us to extract sub-networks which exhibit different properties from the aggregate properties of the whole network and also to investigate information on groups of nodes with similar characteristics which are more likely to be connected to each other. Communities are usually defined as subsets of actors (nodes) that are densely connected, i.e., they are more connected among themselves than to the rest of the network. However, in many network applications, there is a meaningful group structure which does not coincide with the partition into dense communities: indeed, the groups may be characterized by similar patterns of interactions with other groups [5]. Within this context, *positional analysis* is particularly interesting since it deals with the identification of actors who occupy an equivalent position inside a system, i.e. play a similar role in the considered organization. Differently to the standard community detection where the clusters are represented by densely connected groups of actors, positional analysis aims at studying relational data in order to cluster the actors into some classes such that the elements of the same class occupy equivalent positions in the system. In order to illustrate the distinction between positional analysis and standard community detection, let us consider the following example of the e-mails sent among the employees of a company: it may be that we are able to identify different communities of individuals among which e-mails are more frequently exchanged. However, densely connected employees may occupy different positions in the organization and we need to run a positional analysis if we are interested in identifying groups of actors with equivalent positions.

In this work we aim at identifying clusters of “positional equivalent” actors in cases where the available data are the relationships defined among actors on one side and some features on the other one [6], [7], [8], instead of interpersonal relationships. Basically, the idea is that positions in a network structure can be defined according to the characteristics or behaviours that the actors exhibit, instead of the relationships that actors hold with other actors. Individual to attribute relations can be represented as a *weighted bipartite network* where the edge-weights represent the level to which actors show a particular feature. More precisely, a network is bipartite if its nodes can be divided into two sets in such a way that every edge connects a node in one set to a node in the other one [9]. Bipartite networks are thus very useful for representing data in which the elements under scrutiny belong to two categories (typically referred to as actors, or agents, and features, respectively), and we want to understand how the elements in one category are associated with those in the other one. Notable examples that have been analyzed include networks of company directors and the board of directors on which they sit [10], [11], scientific collaboration networks [12], [13], [4], networks of documents and words [14], as well as network of genes and genetic sequences [15]. Models generating bipartite networks can be found also in statistical mechanics (e.g. [16] and [17]).

The widespread approach to partition bipartite networks consists of applying standard community detection algorithms, such as the Girvan-Newman modularity [3], to the one-mode projection of the original network. Consider two types of nodes, say *a* and *b*, in a one-mode projection of the bipartite network, nodes of the same type, say *a*, are connected to each other if they share a common node of the other type, say *b*. For instance, in the CEO network, two CEOs are connected if they both sit in the same board. Although the one-mode projection procedure can give some insights on the topological properties of the network, at the same time it can imply the lost of relevant information. In fact, different bipartite networks may reduce to the same one-mode projection, and thus a clustering based on the latter may produce unreliable or incorrect results, as shown in [18] and [19]. Regardless of those critiques, in [20] the authors argue that under some circumstances, using multiple projections, the information extracted with this procedure is sound, and therefore the simplicity of this approach can be still exploited. However, several authors tried to solve this problem by defining measures and algorithms that could be directly applied to the original matrix associated to the bipartite network.

In the physics community, two different definitions of bipartite modularity have been proposed, [21], [22]. Both concepts extend the Girvan-Newman modularity, but pose different assumptions on the null model taken as the benchmark in the metric used for the module identification. They return good results compared to the one-mode projection, but their applicability is restricted to the case of binary bipartite networks.

Some applications of bipartite networks refer to affiliation networks [23], which capture social relationships, such as membership or event participation. Positional analysis is well established in social network literature, where the usual approach consists of applying the standard measures of structural or regular equivalence, and the related algorithms, to the one mode-projection of the affiliation network [24]. However, affiliation networks represent only a very special case of bipartite networks since the associated matrices are binary.

Other proposed methods for bipartite network clustering, that are mostly used by sociologists, are based on blockmodeling (e.g. [25], [26], [27] and [28]). The key idea of this approach is that the rows and the columns of the matrix associated to the bipartite network can be partitioned simultaneously by means of a criterion function, which measures the inconsistencies of the empirical blocks with the ideal ones. Therefore, blockmodeling works directly on the matrix by trying to permute rows and columns in order to fit, as closely as possible, idealized pictures. The differences between the various types of blockmodeling techniques concern the definition of the ideal blocks and the criterion functions. Blockmodeling is mostly applied to binary data, but it can also be exploited for weighted matrices (valued blockmodeling and homogeneity blockmodeling [28]). However, with the valued blockmodeling, information about the values above a pre-specified parameter is lost and a problem is to determine appropriately the value of this parameter. The homogeneity blockmodeling does not require any additional parameters to be set in advance and it uses all available information, but its main disadvantage is that it can consider only a few possible ideal blocks.

In [29], the authors proposed a bipartite stochastic block model where a parametric probabilistic structure is given, and the clusters are identified by solving the inference problem of finding the parameters that best fit the observed network. In particular, they model the generating process of the number of edges between two nodes of different types with a Poisson distribution with a certain intensity parameter. The authors show that their method outperforms the one-mode projection approach. Nevertheless, it does not deal with the case when we have weights on the edges. In [30], the authors try to go in this direction by proposing a stochastic block model for edge-weighted networks, but their method requires to choose the number of clusters (as in most stochastic block models).

The algorithm we propose realizes a partition of “positional equivalent” actors based on the entire information enclosed in the weighted bipartite network that describes their characteristics or behaviours. The main contribution of our work is twofold. First, we develop a methodological approach according to which actors are grouped with respect to their intrinsic multivariate stochastic dependence structure. In this framework, not only the magnitude of a single weight matters but the whole pattern of the values the actors show along all the features is relevant for the classification. Second, we propose a new clustering procedure that exploits the potentiality of copula functions, a mathematical instrument for the modelization of the multivariate stochastic dependence structure. In particular, copulas allow us to group actors according to their underlying dependence structure, without any assumption on their one-dimensional marginal distributions, and to take into account various kinds of stochastic dependence structures among actors. Moreover, there is no need to predefine the target number of clusters.

The paper is structured as follows. In Sections 1 and 2, we describe our approach, together with the mathematical tool we employ, and we illustrate an algorithm whose output is the exact solution of the optimization problem resulted by our clustering procedure. In Sections 3 and 4 we show the performance of our clustering algorithm applying it to simulated and real data. Finally, in Section 5 we conclude with a discussion on the potentiality of our method and give some heuristics that can be exploited to develop new versions of the algorithm that return “approximate” solutions but are computationally faster.

## A Copula-Based Approach

As explained in the previous section, we consider the general setting where we have an *N*×*M* real-valued matrix, that collects the information on the connections that go from a set of *N* actors to a set of *M* items, representing some features or behaviours. The elements of such a matrix can be any real numbers, with zero representing the absence of a relationship and a non-zero value representing the presence of a relationships, together with its intensity. As an example, this framework can be used to analyse situations where we have actors on one side and personal qualities or interests on the other side, and the weighted-edges between the two sets can be used to represents the level to which an individual shows a certain quality or interest. Another example may be a set of individuals in a supermarket and the set of products they buy. In this case, an edge represent whether an individual bought a particular product or not, and its value gives the amount of product bought or its cost.

Against this background, we want to emphasize that actors may be classified into positions based on their patterns of characteristics, interests or behaviours that they exhibit and on the intensity wherewith the actors show them, instead of the kind of relationships that they keep with other actors. In other words, we move in the direction that the dependence (we mean positive dependence, i.e. similarity) in the expression levels of the considered features is related to the position that the actors occupy in the system. Hence, we say that some actors are *positional equivalent* if they show a significant dependence structure that join them. In this framework, the use of the traditional one-mode projection methods would be meaningless and misleading and also blockmodeling or modularity approaches adapted to bipartite networks could not give a clear answer to the problem because they are not well tailor made for the analysis of weighted bipartite networks.

Our purpose is to identify clusters of actors by means of the detection, from the original matrix, of some statistically significant dependencies among groups of actors. Basically, our assumption is that actors within a system have an underlying multivariate stochastic dependence structure which generates the data. In order to identify this intrinsic dependence structure, we propose to exploit the mathematical copula theory.

The concept of copula was introduced during the forties and the fifties with Hoeffding [31] and Sklar [32], but the evidence of a growing interest in this kind of functions in statistics started only in the nineties [33]. Copulas are functions that join or “couple” multivariate distribution functions to their one-dimensional marginal distributions. More precisely, we have the following definition and results.

**Definition 1.** *A d-dimensional copula C*(**u**) = *C(u_1_,…,u_d_) is a function defined on* [0, 1]^*d*^ *with values in* [0, 1], *which satisfies the following three properties:*

*1.* 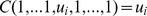 *for every* 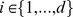 *and* 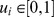;

*2. if u_i_ = 0 for at least one i, then C(u_1_*,…,*u_d_) = 0*;

*3. for every* 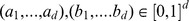 *with a_i_*≤*b_i_ for all i*, 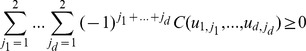

*where, for each i, u_i,1_* = *a_i_ and u_i,2_* = *b_i_*.

The advantage of the copula functions and the reason why they are used in the dependence modeling is related to the Sklar's theorem [32]. It essentially states that every multivariate cumulative distribution function can be rewritten in terms of the margins, i.e. the marginal cumulative distribution functions, and a copula.

**Theorem 1.** *Let F be a multivariate cumulative distribution function with margins* 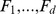. *Then there exists a copula* 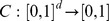 *such that, for every* 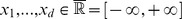, *we have*

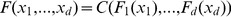
(1)


*If the margins F*_1_,…,*F_d_ are all continuous, then C is unique; otherwise C is uniquely determined on* 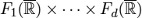.

*Conversely, if C is a copula and F*_1_,…,*F_d_ are cumulative distribution functions, then F defined by (1) is a multivariate cumulative distribution function with margins F*_1_,…,*F_d_*.

In the case when *f* and *f*_1_,…,*f_d_* are the marginal probability density functions associated to *F* and *F*_1_,…,*F_d_*, respectively, the copula density *c* satisfies 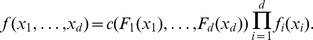



There are different families of copula functions that capture different aspects of the dependence structure: positive and negative dependence, symmetry, heaviness of tail dependence and so on. In our work, we limit ourselves to the principal copula functions of the Archimedean family (namely, Gumbel, Clayton and Frank copulas, see Text S1 for their definitions), which model, through a unique parameter *θ*, situations with different degrees of dependence. Nonetheless, it is worth to note that the application of our methodology is not restricted to those copula functions.

For more details on copula theory, we refer to the various excellent monographs existing in literature, such as [34], [33] and [35].

## Methodology

In this section we present a copula-based technique that realizes a partition of actors into clusters so that the actors belonging to the same cluster show a significant dependence structure that allows us to classify them as being “positional equivalent”. Our approach is inspired by the work of Di Lascio and Giannerini [36], which introduced and studied a copula-based clustering algorithm, called CoClust, in the framework of microarray data in genetics. As they did, we use copula functions in order to model the multivariate stochastic dependence structure among groups of actors and we apply the maximized log-likelihood function criterion for the detection of the different clusters. Notwithstanding, our algorithm presents the following important differences with respect to the one proposed by Di Lascio and Giannerini:

1. while they assume independence within clusters and dependence between clusters, we look for clusters of dependent actors;
2. while they first find the optimal number *K* of clusters and then perform sequential extractions of *K* actors, where at each time one actor is added to each cluster in a certain way, we do not use a sequential extraction method but we directly look for the optimal partition of the actors into clusters;
3. differently from them, we allow clusters to be of different sizes and we allocate all the actors into the clusters;
4. whereas they assume identity in distribution for actors inside a certain cluster, i.e. each cluster identifies one margin, we do not make this assumption and we estimate for each actor his own cumulative distribution function.

Given *N* actors and *M* items, we can represent the data that describe the relationships between actors and items with a real-valued matrix *X* of dimension *N*×*M*, 
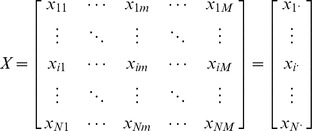
where *x_im_* represents the value of the item *m* for the actor *i* and 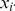 is the row-vector that contains all the item values of the actor *i*. With the language of network theory, this matrix can be seen as the matrix associated to a weighted bipartite network.

The procedure we propose takes as input this matrix and returns the optimal decomposition into clusters after the following four steps:

1. It derives the margin for each actor *i* by finding the empirical cumulative distribution function 
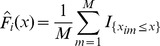
based on the corresponding *M*-dimensional row 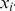 of the items. For each actor *i*, we are taking the values 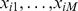 of the *M* items as i.i.d. realizations drawn from the same univariate distribution.

2. It considers each possible cluster 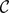 of actors, with 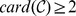, and it computes the maximum value of the copula log-likelihood associated to it. Formally, for each possible group, say 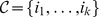, with 2≤*k*≤*N*, of actors, it maximizes the copula log-likelihood function defined as 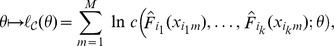
where 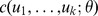 denotes the parametric expression of the density for the chosen copula, and it records the value 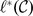 such that 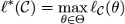



Note that we are taking the vectors 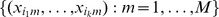 as *M* i.i.d. realizations drawn from the same *k*-variate distribution.

3. It considers the set 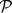 of all possible partitions of the *N* actors that do not contain clusters with a single actor. Hence, each 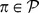 is formed by a certain number of clusters 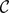 with 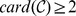. The set 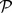 represents the set of all possible decompositions into clusters that the procedure can return. For example, if we have 4 actors, numbered from 1 to 4, the set 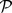 is formed by the following partitions: 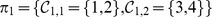, 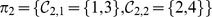, 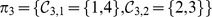 and 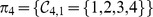. For each 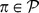, it computes the value of the “global log-likelihood” of the partition *π* as 
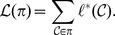



4. Finally, the procedure returns *π* ^*^ with the highest “global log-likelihood” value among all 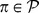, that is 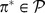 such that 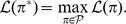



More precisely, it returns the clusters that form *π* ^*^ in a decreasing order with respect to the value 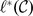 of each cluster 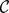 in *π* ^*^.

The R code for this procedure is available at http://dx.doi.org/10.6084/m9.figshare.1162514.

## Simulation Experiments

In order to verify the accuracy of the proposed algorithm, we conducted some simulation experiments by generating random weighted bipartite networks (i.e. their corresponding matrices *X*) by means of copula functions in order to create a clustering structure. In practice, when simulating from a copula of dimension *d*, we obtain *d* vectors 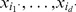 of *M* values that correspond to *d* rows of the matrix *X* associated to *d* actors forming a specific cluster.

An experiment foresees the generation of 50 random weighted bipartite networks of *N* = 10 actors belonging to 3 different clusters. Table 1 reports the 9 scenarios under which we generated these networks. We repeated the simulations of these different scenarios for *M* = 20, 50, 100, 250, thus developing a total of 36 experiments. These experiments were built with the purpose of pointing out different features of the algorithm: first, its performance under different number of items (this is the reason why we used several values of *M*); second, its ability to work with both continuous and discrete data (that explains the choice of the marginal distributions); third, its ability to detect clusters under similar dependence structures (hence we used the same copula type to generate different clusters).

**Table 1:** Description of the scenarios used in the simulation experiment.

S	First cluster	Second cluster	Third cluster
1	*d* = 3, Gu, N(0,1), *θ* = 4	*d* = 4, Gu, N(0,1), *θ* = 3	*d* = 3, Gu, N(0,1), *θ* = 4
2	*d* = 3, Cl, N(0,1), *θ* = 4	*d* = 4, Cl, N(0,1), *θ* = 3	*d* = 3, Cl, N(0,1), *θ* = 4
3	*d* = 3, Fr, N(0,1), *θ* = 4	*d* = 4, Fr, N(0,1), *θ* = 3	*d* = 3, Fr, N(0,1), *θ* = 4
4	*d* = 3, Gu, Po(4), *θ* = 4	*d* = 4, Gu, Po(4), *θ* = 3	*d* = 3, Gu, Po(4), *θ* = 4
5	*d* = 3, Cl, Po(4), *θ* = 4	*d* = 4, Cl, Po(4), *θ* = 3	*d* = 3, Cl, Po(4), *θ* = 4
6	*d* = 3, Fr, Po(4), *θ* = 4	*d* = 4, Fr, Po(4), *θ* = 3	*d* = 3, Fr, Po(4), *θ* = 4
7	*d* = 3, Gu, Pa(1,2), *θ* = 4	*d* = 4, Gu, Exp(0.5), *θ* = 3	*d* = 3, Gu, LogN(0,1), *θ* = 4
8	*d* = 3, Cl, Pa(1,2), *θ* = 4	*d* = 4, Cl, Exp(0.5), *θ* = 3	*d* = 3, Cl, LogN(0,1), *θ* = 4
9	*d* = 3, Fr, Pa(1,2), *θ* = 4	*d* = 4, Fr, Exp(0.5), *θ* = 3	*d* = 3, Fr, LogN(0,1), *θ* = 4

For each scenario (*S*) and each cluster, we report the number of actors in the cluster (*d*), the copula type (Gumbel (Gu), Clayton (Cl), Frank (Fr)), the margins (Normal (N), Poisson (Po), Pareto (Pa), Exponential (Exp), LogNormal (LogN)) and the dependence parameter *θ*, used to generate the data.

As to the copula function employed in the clustering procedure, we first run the algorithm with the same copula used to generate the random network. After that, to test the performance of the algorithm under “misspecification”, we generate again the first three scenarios for all the chosen values of *M* and run the algorithm with the two Archimedean copulas different from the one used in the simulation. For each of the described experiments, we checked the performance of the algorithm by counting the number of times it correctly recognizes the true clusters over the number of random networks generated. Summarizing the results, we observed that the choice of the copula in the algorithm has no great effect on its performance and the overall results seem quite good, especially in the case when *M* = 100 or *M* = 250. Some main remarks can be made:

- First of all, under all the possible scenarios, for *M* = 100 or *M* = 250, we always got a 100% percentage of successes in recognizing the clusters correctly.
- Second, when the observations are drawn from the Gumbel and the Clayton copulas, we got a percentage of successes equal to 100% already for *M* = 50 and between 80% and 100% for *M* = 20.
- Finally, when the observations are drawn from the Frank copula, we notice some problems for *M* = 20. Indeed, for this copula type, 20 realizations are too few to generate an evident dependence structure and so the algorithm does not work well in recognized it. However, we observed a fast improvement for *M* getting larger and, starting from *M* = 50, we can say that the percentage of successes are good (75–80%).

## Empirical Results

In this section, we describe two applications of our algorithm to real datasets. The first one deals with a benchmark real-valued bipartite network that we built on our own to test the algorithm. The second one refers to a widely studied social network that is described by a signed network.

### Trade data

The first application we show is based on the BACI-COMTRADE dataset, featuring the amounts of import-export trades among several countries in the world. We extracted a *weighted bipartite network* taking the export dollar values for the *M* = 97 product categories of the HS2 classification, for selected *N* = 12 countries, in the year 2011. More in details, we decided to select the countries according to their economies, in order to identify 3 hypothetical categories:

- a *First world* category composed by France, Germany, Canada and United states;
- a *Third world* category represented by Burundi, Zimbabwe, Liberia and Somalia;
- an *OPEC representative* category made by Kuwait, Saudi Arabia, Qatar and Iran.

We applied our procedure to the matrix, where the countries were in rows, the products in columns, and each cell contained the gross export value of a given country for a given product. Our aim was to create clusters of countries which are similar (i.e. positional equivalent in the International Trade Network) with respect to the products they export. Much of the literature that focuses on international trade looks for community detection, that is for communities of countries with a high number of connections among them, while being relatively less interconnected with countries outside the community they are part of [37], [38], [39], [40], [41]. Differently from the classical clustering analysis in international trade, we tried to define “positional equivalent” countries based on the products they trade and not on the basis of the countries wherewith they trade. Indeed, we were not interested in finding dense communities of countries for different commodities, but we wanted to identify countries that cover the same position in the trade network since they present a similarity in their exports. The result we obtained is reported in Table 2, together with the results provided by two other clustering methods:

**Table 2:** Trade data.

Our approach			Modularity		K-means		
France	Iran	Burundi	Canada	France	Germany	Burundi	Sau. Ara.
Germany	Kuwait	Somalia	Liberia	USA	USA	France	
USA	Sau. Ara.	Zimbabwe	Sau. Ara.	Zimbabwe		Iran	
Canada	Qatar	Liberia	Iran	Germany		Kuwait	
			Kuwait	Somalia		Somalia	
			Qatar	Burundi		Zimbabwe	
						Qatar	
						Canada	
						Liberia

- *(Modularity optimization)* We derived a unipartite projection of the original weighted bipartite network using a cosine similarity measure between couples of countries and then we applied the modularity optimization approach with the well-known Louvain method [42].
- (*K-means*) with the number of clusters *K* chosen *a priori* equal to 3, see [43]. We use the *K*-means function of the stats R-package.

**Figure 1:**
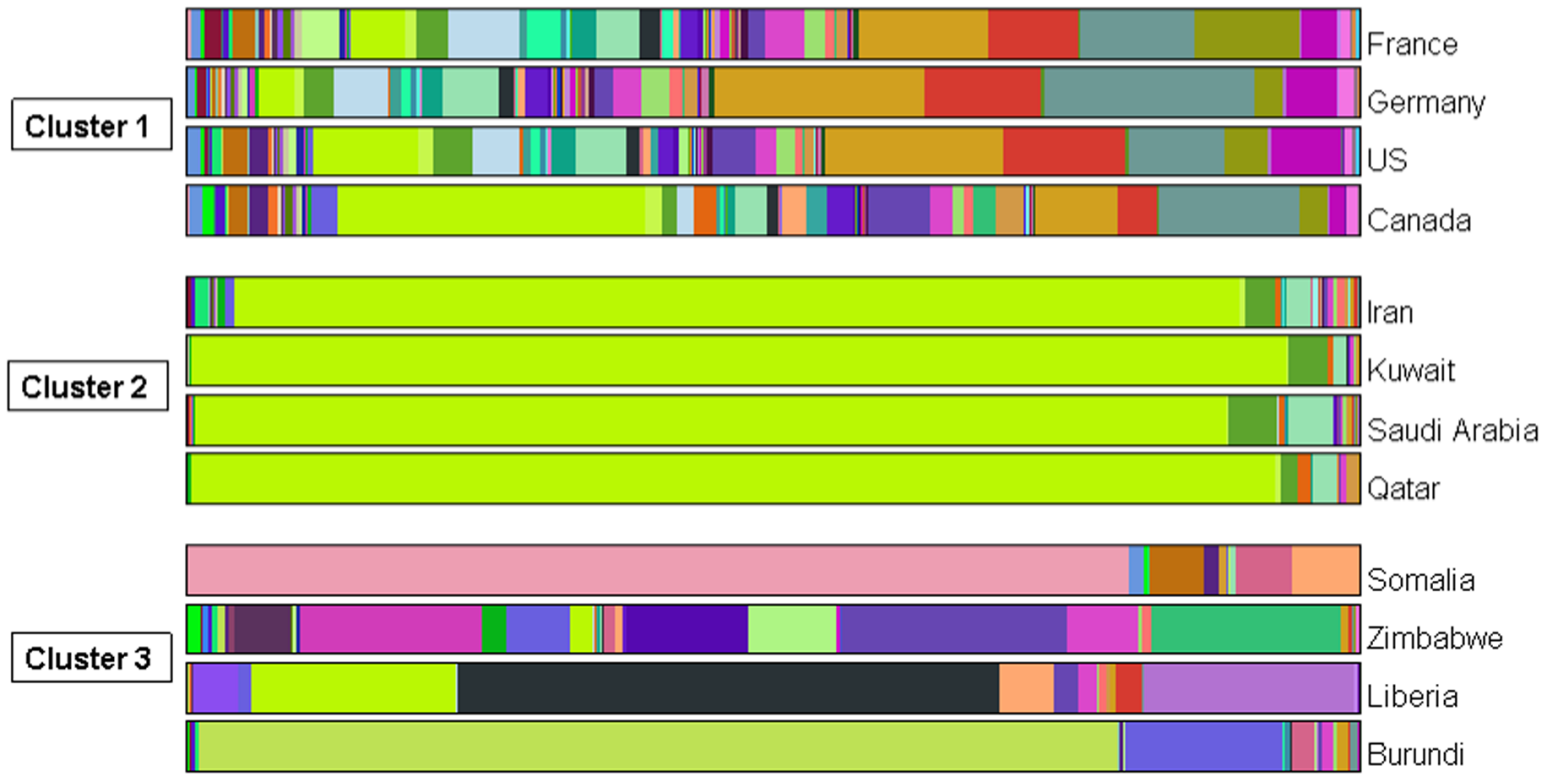
Trade share plot. In this figure we report for each country, classified in the relative cluster, a coloured bar representing the share of export for each of the 97 HS2 product categories over the total amount of export. Regarding *Cluster 1*, the high number of colours into the bar makes it clear that these countries use to trade in several product categories. Furthermore, an explicit dependence pattern arise from the proportion of the colours into the bars. In particular, the following product categories contributes to this strong relationship: **84**, **87**, **88**, **85**, **30**, **90**. Regarding *Cluster 2*, the dependence relationship mainly arises from these three categories: **27**, **29**, **39**. However, it is important to remark that our clustering approach takes in consideration also the fact that these countries trade in a very small number of products, as can be seen from the few colours in the respective bars. The same reasoning apply for *Cluster 3* where, although the countries are specialized in a unique product such as category **9** for Burundi or category **1** for Somalia, the common pattern that makes them similar is the fact that they do not trade in most of the 97 HS2 categories.

**Figure 2:**
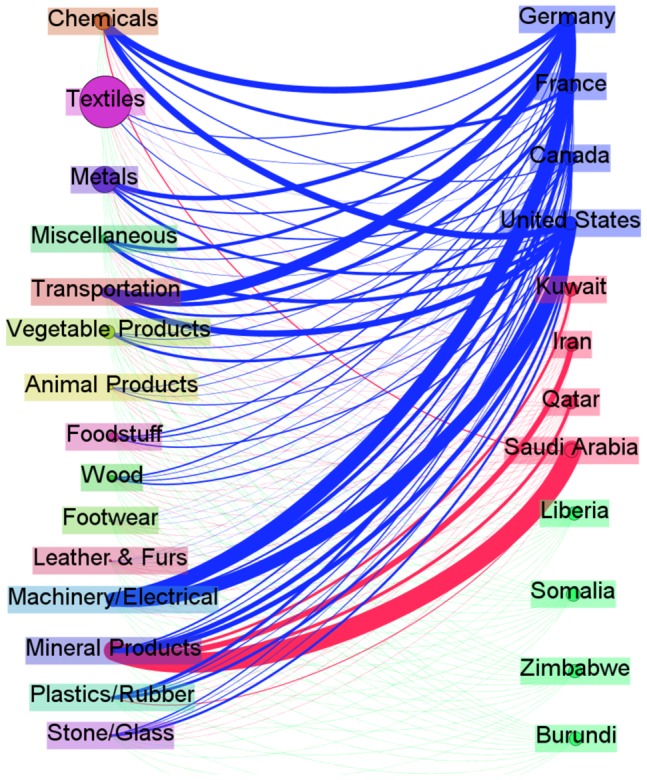
Trade network structure. In this figure we show the weighted bipartite Trade network. On the right the three groups of countries, detected by our algorithm, and on the left the 97 products categories, grouped in 15 homogeneus macro categories in order to highlight the relevant connections among the two different type of nodes. The size of the macro categories are in proportion to the number of categories grouped in them. It is clear from the links partition how our metodology is able to disentangle different country categories according to the trade patterns, even for the third world countries (green background) for which the link weights are much smaller than the others.

As we can see, our algorithm is able to perfectly recognize the above mentioned country groups; while the other two methods provide a different grouping. Since the hypothetical three groups were built according to a subjective judgement, we decided to analyze the data in order to provide a more robust explanation for the clusters we found. An overview of the differences between the three groups is provided by Figure 1, where we report for each country a coloured bar with the export shares for each of the 97 HS2 product categories over the total amount of export. In more detail, in Table 3 we report for each country, the percentage on the total amount of export for a selection of 21 HS2 categories out of the 97 available, in order to give some hints on the trade joint patterns that our algorithm recognize. Overall, we can agree on the fact that the result is coherent with the observed data. Regarding the *First world* category, we can see that at least a small amount of their total exports is allocated in each selected categories and about the 60% of their total export is concentrated in the nine categories, corresponding to the following commodities: **84** - Nuclear reactors, Boilers, Machinery and mechanicals appliances; **87** - Vehicles; **88** - Aircraft and Spacecraft; **85** - Electrical machinery, Telecommunications equipment, Sound and Television recorders; **30** - Pharmaceutical products; **90** - Optical, Photographic, Cinematographic, Measuring, Checking, Precision, Medical instruments. Conversely, for the *OPEC Representative* group, it is clear that the nature of the dependence arises from the fact that more than the 90% of the total export of these countries belongs to the following three categories: **27** - Mineral, Fuels, Oils; **29** - Organic chemicals; **39** - Plastic and Articles thereof. Nonetheless, we underline that our algorithm did not recognize this cluster just because of the large share of export these countries have in these few products, but it captured the whole dependence between these countries and so also the categories in which they do not trade, or trade a little, play an important role. This is clear by looking at the network structure for the *Third world* category in the last four columns of Table 3. As it can be seen, all these countries present a huge amount of the total export in a few specific commodities. For example, more than the 80% of the somalian export is in category **1** - Live animals, while the 78% of the burundian export is in category **9** - Coffee, Tea, Mate and Spices. Thus, we can affirm that these countries present a highly specific production and the dependence among them arise not as a consequence of the products in which they trade but rather from the products in which they do not trade. By looking carefully at Table 3, it is possible to notice that for most of the selected 21 HS2 categories, the share of export is almost zero in all these *Third World* countries. In this sense, they are similar to the *OPEC representative* countries but, as we already said, the latter present a specific dependence deriving from the common commodities they trade. Finally, *Canada* deserves some comments. It has an high value in category **27** as the countries in the *Opec representative* category, but its values for the other categories are more similar to those of the *First world* than the ones of the *Opec representative* group. Our algorithm is able to capture this aspect. An insight of all these distinguishing features between the clusters can also be grasped looking at Figure 2, where we depict the bipartite trade network between the countries and 15 macro-categories of the HS2 products classification.

**Table 3:** Selected sample of 21 HS2 products traded by each country.

HS2	Countries											
	France	Germany	U.S.A	Canada	Iran	Kuwait	Saudi Arabia	Qatar	Somalia	Zimbabwe	Liberia	Burundi
84	11.14	17.83	15.20	7.05	0.41	0.09	0.29	0.11	0.04	0.57	0.54	0.86
87	9.74	17.86	8.22	12.07	0.21	0.06	0.17	0.01	0.00	0.15	0.06	0.69
88	8.86	2.41	3.63	2.34	0.02	0.04	0.08	0.00	0.00	0.12	0.00	0.00
85	7.60	9.85	10.38	3.34	0.22	0.05	0.21	0.03	0.02	0.36	2.27	0.21
30	6.06	4.62	4.01	1.44	0.10	0.03	0.05	0.01	0.00	0.13	0.04	0.02
90	2.97	4.34	5.96	1.29	0.05	0.01	0.03	0.01	0.00	0.02	0.04	0.10
27	4.64	3.08	9.02	26.22	85.69	93.33	87.79	92.33	0.00	1.87	17.29	0.00
39	3.70	4.84	4.36	2.82	2.02	1.19	3.84	2.09	0.52	0.20	0.09	0.03
29	2.65	2.47	3.38	1.32	2.53	3.38	4.19	1.50	0.00	0.02	0.10	0.00
31	0.09	0.27	0.39	1.96	0.50	0.43	0.45	1.03	0.00	0.21	0.00	0.00
1	0.43	0.12	0.08	0.33	0.03	0.01	0.03	0.01	80.23	0.04	0.00	0.05
9	0.06	0.19	0.07	0.11	0.14	0.00	0.01	0.00	0.00	0.58	0.15	78.11
71	1.38	1.56	3.68	5.31	0.34	0.03	0.20	0.15	0.01	19.09	2.10	0.29
40	1.66	1.31	1.14	0.93	0.05	0.09	0.01	0.00	0.00	0.23	46.07	0.04
97	0.45	0.07	0.36	0.05	0.01	0.00	0.00	0.02	0.00	0.11	0.07	0.00
96	0.24	0.17	0.10	0.02	0.01	0.00	0.00	0.00	0.00	0.03	0.00	0.02
95	0.26	0.39	0.34	0.23	0.00	0.00	0.00	0.00	0.00	0.03	0.00	0.00
93	0.07	0.06	0.22	0.05	0.00	0.00	0.00	0.00	0.00	0.00	0.20	0.00
92	0.04	0.05	0.05	0.02	0.00	0.00	0.00	0.00	0.00	0.00	0.00	0.02
91	0.28	0.11	0.06	0.01	0.00	0.01	0.00	0.02	0.00	0.00	0.22	0.00
86	0.20	0.36	0.23	0.10	0.00	0.00	0.00	0.00	0.00	0.02	0.01	0.00

The table contains the percentage on the total amount of export for some selected product categories; while we applied the algorithm directly on the export values for all the 97 categories.

### Supreme Court voting data

The second application is based on the dataset used in [27] of the Supreme Court judges and their votes on a set of issues. We have a *signed bipartite network* [44] with *N* = 9 justices, *M* = 26 issues and the expressed votes.

In Table 4, we present both our result and the one in Doreian [27]. Although the number of clusters is different, we notice that the two approaches classify, exactly in the same way, the first two members of the first cluster and those ones of the second cluster. Contrarily, a remarkable difference stems from the fact that our algorithm groups together *Kennedy*, *O'Connor* and *Rehnquist* while Doreian [27] put them in three different clusters (two of which have a single element). Regarding this, we need to recall that our algorithm does not allow for the size of the cluster to be lower than two, thus the third cluster arises as a residual one.

**Table 4:** Justice data.

Our method			Doreian [27]			
Cluster 1	Cluster 2	Cluster 3	Cluster 1	Cluster 2	Cluster 3	Cluster 4
Scalia	Breyer	Kennedy	Scalia	Breyer	Kennedy	O'Connor
Thomas	Ginsburg	O'Connor	Thomas	Ginsburg		
	Souter	Rehnquist	Rehnquist	Souter		
	Stevens			Stevens		

**Figure 3:**
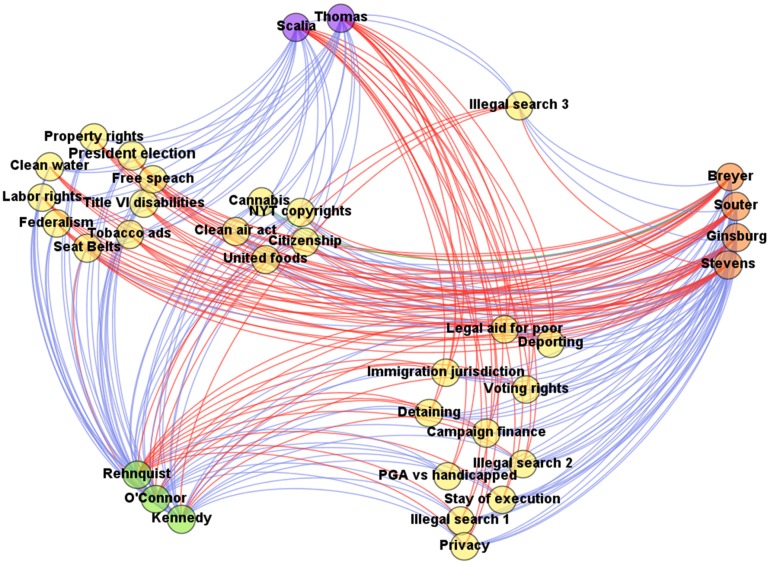
Justice sentences network. This figure depicts the bipartite signed network of the US Supreme Court Justice votes upon 26 different issues. The blue edges correspond to votes in the majority (+1), the red edges correspond to votes in the minority (−1) and the unique green edge correspond to a case of abstention (0). Furthermore, the nodes are classified as follow: yellow for the 26 issues, violet for *Cluster 1*, orange for *Cluster 2* and green for *Cluster 3*. The network has been built so as to capture the sharpness of the clusters partitioning. In particular, an higher cohesiveness among the judges within the first and second clusters with respect to those ones in the third cluster can be ascertained by the fact that a more coherent coloured pattern can be glimpsed from the beam of edges that originate from the first two clusters with respect to the last one, i.e. two different stacks can be distinguished, a red one and a blue one.

Next to these first considerations, it is interesting to deepen the analysis by studying the data structure and try to give a more detailed explanation for the differences. To this end, we report in Table 5 a permuted version of the Supreme Court voting matrix, where the issues are blocked as in [25] and the judges are partitioned according to the results from our algorithm, whereas in Figure 3 we depict the bipartite network structure. Looking at the first cluster, containing *Scalia* and *Thomas*, and the second cluster, composed by *Breyer*, *Ginsburg*, *Souter*, and *Stevens*, we can easily recognize a voting pattern remarkably opposed one to each other and at the same time a coherent preference expression within the groups.

**Table 5:** Supreme Court voting data.

Issue (*M* = 26)	Supreme Court justice (*N* = 9)								
	Br	Gi	St	So	OC	Ke	Re	Sc	Th
President election	−1	−1	−1	−1	1	1	1	1	1
Federalism	−1	−1	−1	−1	1	1	1	1	1
Clean Water	−1	−1	−1	−1	1	1	1	1	1
Title VI Disabilities	−1	−1	−1	−1	1	1	1	1	1
Tobacco Ads	−1	−1	−1	−1	1	1	1	1	1
Labour rights	−1	−1	−1	−1	1	1	1	1	1
Property Rights	−1	−1	−1	−1	1	1	1	1	1
Citizenship	−1	−1	1	−1	−1	1	1	1	1
Free Speech	1	−1	−1	−1	1	1	1	1	1
Seat Belts	−1	−1	−1	1	−1	1	1	1	1
United Foods	−1	−1	1	1	−1	1	1	1	1
NYT Copyright	−1	1	−1	1	1	1	1	1	1
Cannabis for Health	0	1	1	1	1	1	1	1	1
Clean Air Act	1	1	1	1	1	1	1	1	1
PGA vs. Handicapped	1	1	1	1	1	1	1	−1	−1
Illegal Search 3	1	1	−1	1	−1	−1	−1	1	1
Illegal Search 1	1	1	1	1	1	1	−1	−1	−1
Illegal Search 2	1	1	1	1	1	1	−1	−1	−1
Stay of Execution	1	1	1	1	1	1	−1	−1	−1
Privacy	1	1	1	1	1	1	−1	−1	−1
Immigration Jurisdiction	1	1	1	1	−1	1	−1	−1	−1
Detaining Criminal Aliens	1	1	1	1	−1	1	−1	−1	−1
Legal Aid for the Poor	1	1	1	1	−1	1	−1	−1	−1
Voting Rights	1	1	1	1	1	−1	−1	−1	−1
Deporting Criminal Aliens	1	1	1	1	1	−1	−1	−1	−1
Campaign Finance	1	1	1	1	1	−1	−1	−1	−1

The data on the Supreme Court judges can be found in [27]. The blocks of the issues are based on [25]. The table is filled with +1 if the judge voted in the majority for that issue and with −1 if he was in the minority for that decision. In case a 0 is reported, it means that for that particular case the judge refrained to vote.

The unique puzzling doubt concerns the allocation of *Rehnquist* in the group of *Kennedy* and *O'Connor* rather than in the group of *Scalia* and *Thomas*. In order to further investigate this issue, we decided to check the global likelihood value in the case where we move *Rehnquist* in the first group. What we found is that the addition of him to the group of *Scalia* and *Thomas* considerably decreases the global likelihood. This effect is a consequence of the fact that our procedure recognizes the perfect dependence among these last two actors, and therefore it prefers to allocate *Scalia* and *Thomas* alone in one cluster in order to point out their “positional equality”, and to group into the third cluster *O'Connor*, *Kennedy* and *Rehnquist*, which perfectly agree over half of the issues.

## Conclusions and Future Lines of Research

Clustering algorithms have increasingly assumed a central role for the identification of communities in complex networks. In this paper, we deal with a notion of community different from the classical one: while the network clustering analysis, namely the community detection, aims to identify clusters of densely connected actors, we try to determine groups of actors that play a similar role inside a certain organization basing on the characteristics or habits that they exhibit. In the social network literature, this is known as positional analysis.

To this end, we propose a new clustering algorithm that can be applied to situations which are suitably modelled through a *weighted bipartite network*. Starting from the associated real-valued matrix, with the actors on the rows, the features on the columns, and the weights as the elements, we try to capture possible similarities among groups of actors by analyzing the multivariate stochastic dependence among them.

The contribution of this paper has to be found in the methodological approach we propose for positional analysis that is based on the detection of the intrinsic multivariate stochastic dependence among groups of actors and in the development of a new related algorithm that uses copula functions in order to model these dependence structures. Furthermore, this algorithm directly operates on the matrix describing the actor-feature relationships, differently from many other algorithms that collapse the information of the bipartite network to a unipartite one and then apply the classical clustering procedure. In fact, this kind of operation can cause a lost of information and a consequent erroneous cluster identification. Another advantage of our technique is that it finds the optimal partition, without fixing *a priori* the number of clusters and the maximum number of elements per cluster (though we don't allow for cluster of single elements). Furthermore, our algorithm is able to work directly on any matrix, binary or weighted with real numbers.

**Figure 4:**
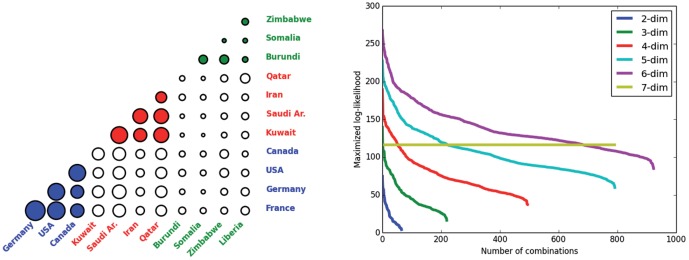
Log-likelihood plots. (Left) For each couple of countries, the figure shows circles whose areas correspond to the maximum log-likelihood values of the bivariate copulas. The colours are used to identify the countries belonging to the same cluster. (Right) For different dimensions of the copula function, the figure shows the maximum log-likelihood values for each possible combination of countries, in decreasing order.

This is the first time this methodology is applied to the network field, therefore it is not surprising that there are still some issues to be addressed, and that we leave for future research. The major drawback of our algorithm concerns the high computational burden it bears as a consequence of the fact that it explores all the possible combinations of groups of actors. Since our first purpose was to understand the potentiality of such a new approach, we have not tried to develop any optimized version of the algorithm yet. For the moment, we have provided an algorithm whose output is the exact solution of the optimization problem, as it explores all the possible combinations. However, we are convinced that a deeper study of its behaviour could give some criteria to reduce the number of combinations to explore and allow the development of a new version of the algorithm that provides an “approximate” solution but is computationally faster. For instance, on the left side of Figure 4 we report the maximum copula log-likelihood values of each couple of countries obtained from the trade example in Section 4. It can be noticed that from the log-likelihood values of the bivariate copulas, we already have some insights on the possible clusters. In fact, we can see that the bivariate copula log-likelihoods of those countries belonging to the same cluster tend to be higher than the others. Given this information, we could for example adopt an agglomerative approach, as it is common in the community detection literature [42], and group those actors that present a more significant bivariate dependence so as to avoid the calculation of all the possible combinations for the various dimensions.

Another heuristic argument we could exploit to reduce the computational cost can be deduce from the right side of Figure 4, where we plot the maximum log-likelihood values of copulas with different dimensions for all the combination of countries used in the trade example in Section 4. We can see that, for this study case, the maximum log-likelihood value of the 7-dim copula is constant across the combinations so as to suggest that clusters larger than six countries are less plausible. Therefore, it seems that the algorithm recognizes some sort of upper bound for the cluster size, and we can exploit this information to avoid all those calculations over the sixth dimension.

A second issue concerning the proposed algorithm consists in that it does not allow for clusters with a single element, and in positional analysis it may be a limitation. We avoid to address this issue because it would cause an increase of computational cost, but it could be theoretically feasible to exploit the copula functions in such a way to consider also clusters of a single element. Regarding this point, we also point out that, since the present algorithm returns the clusters in decreasing order with respect to the maximum copula log-likelihood value, the eventual single elements are contained in the last (residual) cluster, see for instance Table 4.

In conclusion, though there are still some open issues to be solved in order to apply this new clustering algorithm to large networks, it seems to capture dependence patterns that other algorithms ignore. Therefore, we strongly believe it could have interesting implications on positional analysis in the future, and we foster future developments of this approach.

## Supporting Information

Text S1 **Archimedean family of copulas**. Technical description of the copula functions employed in the analysis.
(PDF)
