# Supplementary material for: Cluster Analysis of Weighted Bipartite Networks: A New Copula-Based Approach
Source: PLoS One. 2014 Oct 10;9(10):e109507. doi: 10.1371/journal.pone.0109507 (PMC4193785; doi:10.1371/journal.pone.0109507)
Supplement: Text S1 — Archimedean family of copulas. Technical description of the copula functions employed in the analysis. (PDF) [file pone.0109507.s001.pdf]

Text S1. Archimedean family of copulas

Here we recall just the principal copula functions belonging to the Archimedean family that we employ in our simulations and real data analysis.

The Archimedean family is defined using a specific function ϕ, known as the *generator*, by means of the formula

$$C(\mathbf{u}) = \phi^{-1}(\phi(u_1) + ... + \phi(u_d)).$$

Different functional forms of the generator entail different dependence structures. The principal Archimedean copulas are the following.

- **Gumbel copula**. The generator is given by $\phi(u) = (-\ln(u))^\theta$ and so the Gumbel copula is defined as

$$C^{Gu}(\mathbf{u};\theta) = \exp\left\{ -\left[\sum_{i=1}^{d}(-\ln u_i)^\theta\right]^{\frac{1}{\theta}} \right\}, \qquad \theta \in \Theta = [1, +\infty).$$

  The parameter θ tunes the degree of the dependence. In particular, the value $\theta = 1$ corresponds to independence (indeed, we get $C^{Gu}(\mathbf{u}; 1) = \prod_{i=1}^{d} u_i$).

- **Clayton copula**. The generator is given by $\phi(u) = (u^{-\theta} - 1)/\theta$ and so the Clayton copula is defined as

$$C^{Cl}(\mathbf{u};\theta) = \left[\sum_{i=1}^{d} u_i^{-\theta} - d + 1\right]^{-\frac{1}{\theta}}, \qquad \theta \in \Theta = (0, +\infty).$$

- **Frank copula**. The generator is given by $\phi(u) = -\ln\left(\frac{\exp(-\theta u)-1}{\exp(-\theta)-1}\right)$ and so the Frank copula is defined as

$$C^{Fr}(\mathbf{u};\theta) = -\frac{1}{\theta}\ln\left(1 + \frac{\prod_{i=1}^{d}(\exp(-\theta u_i) - 1)}{(\exp(-\theta) - 1)^{d-1}}\right), \qquad \theta \in \Theta = (0, +\infty).$$

Also for these two last copulas, the parameter θ controls the degree of the dependence.
